# Environmental impact of switching from the synthetic glucocorticoid prednisolone to the natural alkaloid berberine

**DOI:** 10.1371/journal.pone.0199095

**Published:** 2018-06-14

**Authors:** Iris E. Allijn, Rik Oldenkamp, Gert Storm, Ad M. J. Ragas, Raymond M. Schiffelers

**Affiliations:** 1 Department of Biomaterials Science and Technology, TechMed Centre, University of Twente, Enschede, The Netherlands; 2 Department of Environmental Science, Institute for Water and Wetland Research, Radboud University, Nijmegen, The Netherlands; 3 Environment Department, University of York, York, United Kingdom; 4 Department of Pharmaceutics, Utrecht University, Utrecht, The Netherlands; 5 Department of Science, Open Universiteit, Heerlen, The Netherlands; 6 Clinical Chemistry and Haematology, University Medical Centre, Utrecht, The Netherlands; University of Pittsburgh, UNITED STATES

## Abstract

Low amounts of human pharmaceuticals in the aquatic environment can affect bacteria, animals and ultimately humans. Here, the environmental consequences of a shift in prescription behavior from prednisolone to berberine was modeled using an environmental decision support system based on four consecutive steps: emission, fate, exposure and effect. This model estimates the relative aquatic and human health impacts of alternative pharmaceutical prescriptions throughout Europe. Since a Defined Daily Dose (DDD) of berberine has yet to be formulated, the environmental impacts of berberine and prednisolone were compared under the assumption of equal DDDs. Subsequently, the relative impact ratio indicates the extent to which the actual DDD of berberine might be higher to still be environmentally preferable over prednisolone. In fact, berberine can be administered at a six times higher dose throughout Europe before its impact on the aquatic environment exceeds that of one prescription of prednisolone. On average, the results for impacts on human health are similar, with the median impact ratio ranging between 5.87 and 22.8 depending on the level of drinking water purification. However, for some regions in Spain, Austria, Baltic States and Finland, berberine can only be considered an environmentally better alternative if it is administered at a lower dose than prednisolone. We conclude that for most regions in Europe it is, up until a certain dose of berberine, beneficial for the aquatic environment and therefore human health to prefer prescription of berberine over prednisolone.

## Introduction

Low amounts of human pharmaceuticals in the environment, even below the safety and efficacy test concentrations, can affect bacteria and animals [1,2]. Especially in the aquatic environment, there might be an unacceptable risk due to chronic exposure to pharmaceuticals [3]. In particular, one of the main problems caused by (human) pharmaceuticals in the environment is endocrine disruption. This includes disruption of fertility, development of feminized fish, disruption of the normal growth of fish, reptiles and aquatic invertebrates [1,4]. Because of their similarity in structure to endogenous hormones, endocrine disruption is often ascribed to steroidal drugs, but can be caused by non-steroidal compounds as well [5,6]. There is also a potential risk for humans and although the environmental concentrations are generally regarded as safe [1], it is mostly unknown what fraction of pharmaceuticals eventually make their way back to humans [6]. This can be through accumulation in the food chain [1,5,6] or *via* drinking water [2]. The presence of human pharmaceuticals in the environment appears an underestimated problem [1] with unknown consequences [6].

Already in the 1960s, the presence of human pharmaceuticals in the environment was anticipated [7] and proven in the 1980s [6]. It took until the 1990s for water contamination by pharmaceuticals to become an environmental issue [8–10]. In order to halt the potential environmental damage caused by human pharmaceuticals, governments may install laws to restrict their emission into the environment and provide education and return programs [11]. Despite the increased attention for the environmental impact of human pharmaceuticals since the start of the millennium [1], little regulation is currently in place [12]. The major programs that are in place, are the Toxic Substances Control Act (TSCA) [13] of the USA and the Registration, Evaluation, Authorisation and Restriction of Chemicals (REACH) program [14] of the EU. However, the TCSA does not involve environmental risk management for hazardous substances and REACH does not include human pharmaceuticals [12]. Furthermore, the mandatory Environmental Risk Assessment (ERA) from the European Medicines Agency (EMA) [15], says that for all drugs the environmental impact should be assessed: in the first phase, the substance should be pre-screened for consumption data and for the octanol-water partitioning coefficient (*k*_*ow*_). If the log *k*_*ow*_ > 4.5, additional persistence, bioaccumulation and toxicity screenings should be performed. In the second phase, aquatic toxicity, emission and fate should be assessed in a refined risk assessment. However, from these tests the vitamins, electrolytes, amino acids, peptides, proteins, carbohydrates, lipids, vaccines and natural products are exempted. Furthermore, tests results cannot be a criterion for refusal for marketing [15].

To be able to predict the environmental consequences of specific molecules, it is important to have proper fate and effect assessment protocols in operation in not only rats and mice, but also in algae, fish and water fleas. These protocols can help to predict whether a compound can reach hazardous concentrations in the aquatic, terrestrial or atmospheric environment [6,16,17]. This would complement the safety-testing of pharmaceutical companies since these tests are of limited duration and therefore do not mimic the environmental conditions [2]. Up to now, pharmaceutical companies have only to a limited extent included environmental impact in their selection of active pharmaceutical ingredients. Including this element in the development and selection of pharmaceutical ingredients is advocated to make the transition to green pharmacy and chemistry [6,11,18,19].

**Fig 1:**
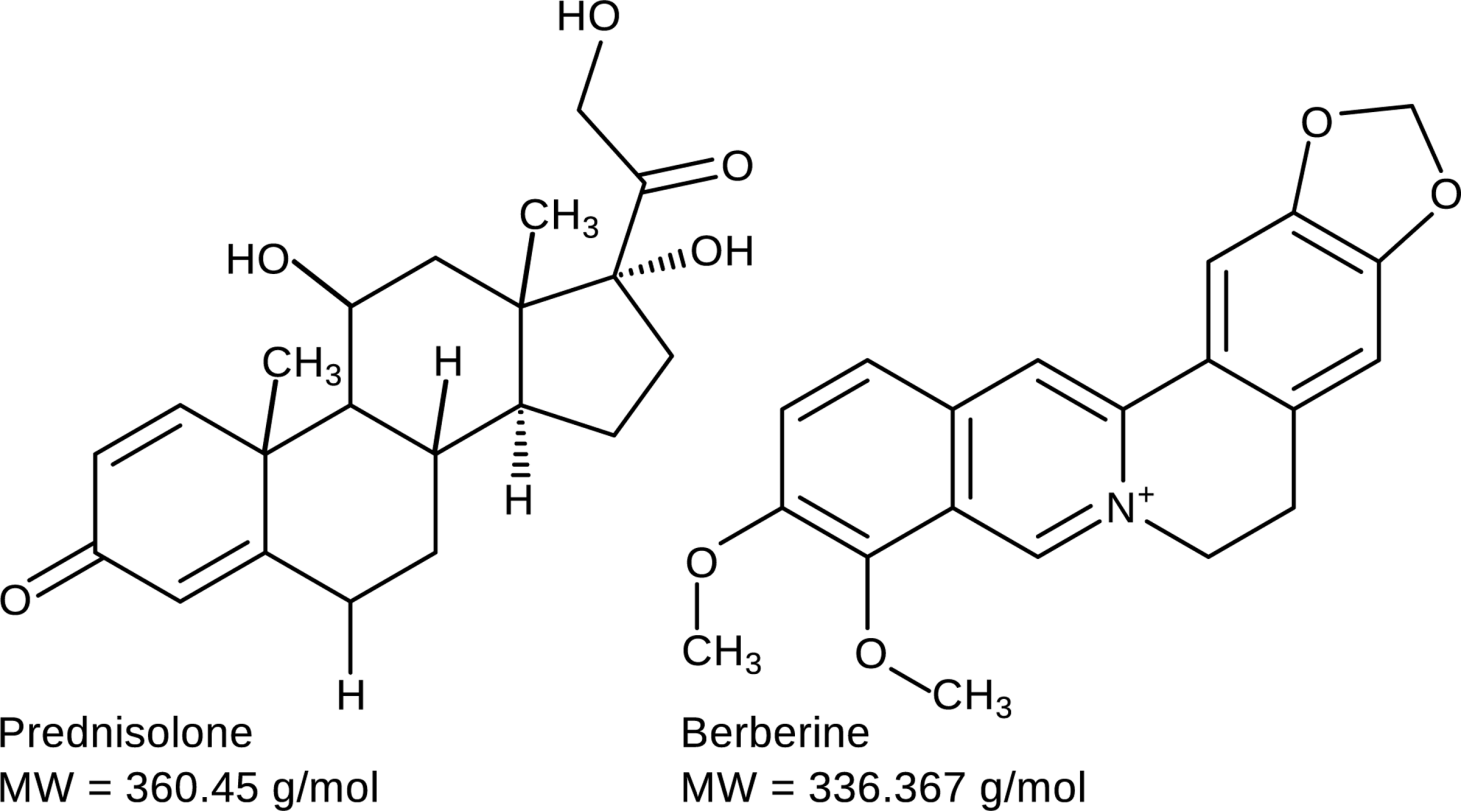
Molecular structures of the synthetic glucocorticoid prednisolone (CAS RN = 50-24-8) and the natural alkaloid berberine (CAS RN = 2086-83-1).

Corticosteroids are the most potent anti-inflammatory agents and are both prescribed as over-the-counter drugs in certain countries as well as prescription-only medication in others. They are extensively used for a variety of conditions. The prescribed dose and administration route depends on the severity of the inflammatory symptoms and medical condition [20,21]. Prednisolone (Fig 1) is a well-known synthetic corticosteroid, mainly prescribed in inflammatory diseases such as rheumatoid arthritis. Available metabolism data of prednisolone show that up to 24% is excreted in its unchanged form [22]. Furthermore, prednisolone is the active metabolite of the synthetic drug prednisone, which is an often used pharmaceutical as well. Altogether, this implies that a substantial amount of prednisolone ends up in the environment [22]. Synthetic steroids are designed to have strongly enhanced potencies compared to natural hormones. As a result, they may be able to induce endocrine disruption in aquatic organisms. But also immune depression and neurobehavioral changes have been reported [23,24].

Natural products are gaining interest because of their potent anti-inflammatory and anti-oxidant properties [25–27]. The therapeutic window of natural products might be larger than for corticosteroids. A promising anti-inflammatory natural product that is currently under investigation is the isoquinoline quarternary alkaloid berberine [28] (Fig 1). It has been shown to reduce inflammation *in vitro via* pathways different than those in case of prednisolone [29]. However, the impact of berberine on the environment has not yet been investigated.

In this study, we aimed to model the environmental consequences of a shift in prescription behavior from the synthetic corticosteroid prednisolone to the natural product berberine. We hypothesized that berberine due to its natural source would have less environmental impact than the synthetic drug prednisolone. Human and aquatic toxicity and fate parameters of the compounds were fed into a model as previously described by Oldenkamp *et al*. [17] and used to assess the environmental impact of pharmaceutical prescriptions throughout Europe. This model was used to describe the relative impacts of berberine and prednisolone on the environment and on human health resulting from environmental exposure.

## Methodology

### Description of the model

**Fig 2:**
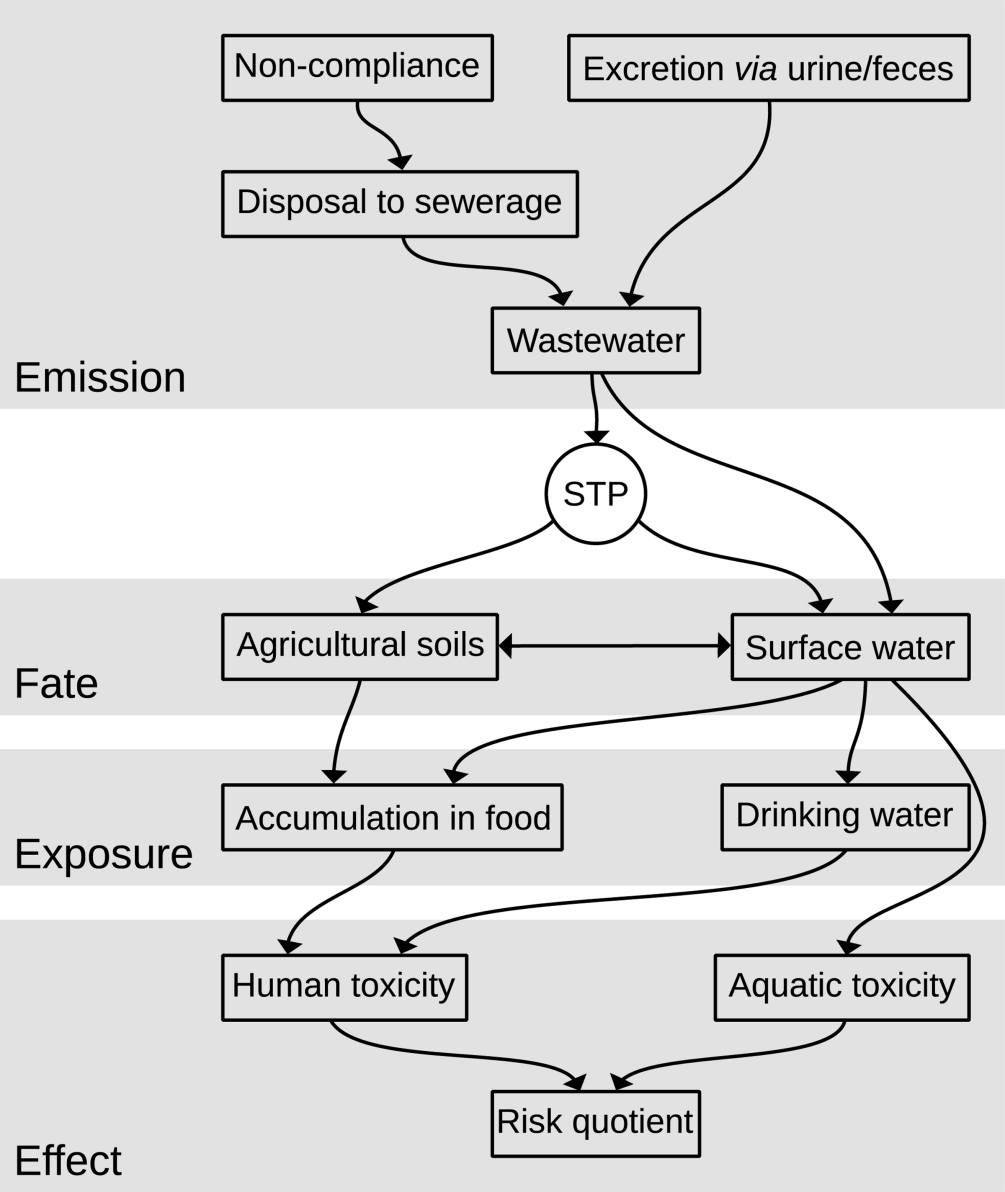
Schematic visualization of the processes used for the calculations. STP = sewage treatment plant. Adjusted from Figure A1 from Oldenkamp *et al*., 2014 [17].

The environmental decision support system described by Oldenkamp *et al*. [17] was used to model the environmental consequences of a postulated shift in prescription behavior from prednisolone to berberine. This methodology was originally developed for the location specific assessment and comparison of the environmental impact of two alternative pharmaceutical prescriptions, aiming to provide physicians with the opportunity to include environmental considerations in their prescription practice. The model provides regionalized estimates of the relative impacts of alternative pharmaceutical prescriptions throughout Europe, for both the aquatic environment and human health. It is based on the four consecutive steps of emission, fate, exposure and effect estimation (Fig 2).

As a first step, emissions into wastewater are calculated as the sum of non-compliance and subsequent disposal *via* flushing, and of actual consumption and excretion as parent compound *via* urine or feces. Pharmaceutical residues in wastewater are estimated at the level of individual Member States, before they are divided over individual agglomerations, based on their population size. Generally, this wastewater is discharged into the surface water after passage through a sewage treatment plant (STP). However, part of the wastewater might also be discharged into the surface water directly, depending on the local level of STP-connectivity. Indirect emissions (*i*.*e*. after passage through an STP) are calculated at the level of the individual STPs, and depend on STP design and active pharmaceutical ingredient (API)-specific removal rates corresponding with the treatment techniques applied. Furthermore, the model also estimates emissions of APIs to agricultural soils, which depend on pharmaceutical levels in secondary sewage sludge and Member State specific sludge disposal practices.

To enable the second step of multimedia fate calculations with the model SimpleBox (*e*.*g*. Hollander *et al*., 2009 [30]), emissions to surface water and agricultural soils are aggregated at the level of 100 * 100 km environmental grid cells, spatially parameterized with data from Pistocchi *et al*. [31]. These calculations result in yearly averaged steady-state surface water and soil concentrations. Relative aquatic risk quotients are calculated as the ratio between these concentrations and API-specific HC_50_ values (*i*.*e*. the concentration at which 50% of the individuals in 50% of the aquatic species are being affected). Finally, these risk quotients are used to derive a grid cell-specific prioritization of the APIs assessed, based on their relative aquatic impacts.

The calculation of relative human health risk quotients in the third step requires additional exposure calculations, including estimations of the transfer of pharmaceutical residues into foodstuffs and drinking water, and age- and location-specific behavioral and consumption patterns. Human contact media taken into account are drinking water, fruits and vegetables, meat products, milk products, fish, surface water, and soil. The degree of exposure is determined by the concentrations in these contact media as well as the intensity of the contact with them. Concentrations in food were estimated from those in surface water and agricultural pore water using bioconcentration factors (BCFs) for fish [32], root concentration factors (RCFs) for fruits and vegetables [33] and biotransfer factors (BTFs) for meat and milk products [34].

Concentrations in drinking water depend on the source of the water and the purification techniques applied. While data on the first were available at the level of the individual EU Member States [35], spatially explicit data on drinking water purification levels were not. The model addresses this by formulating different purification scenarios: conventional, advanced and no treatment. The conventional purification scenario was regarded as the minimum scenario in order for the EU Member States to meet the European quality standards (EU Council Directive 98/83/EC), and it consists of in series application of coagulation, powdered activated carbon (PAC), chlorination and sand filtration. In addition to these techniques, the advanced treatment scenario also includes the application of membrane bioreactor (MBR), UV-treatment, ozonation, reverse osmosis and nanofiltration. The scenario of no treatment was specifically formulated to address small scale groundwater sources and was excluded from the present study.

Then, in the last step, the model derives average daily human exposure estimations for a range of exposure groups, characterized by age, nationality and other factors (*e*.*g*. drinking water purification level and food origin). In a previous study, infants (0–1 years) that consumed locally produced foodstuffs were identified as the most sensitive human exposure group [16]. Therefore, we selected them as the human exposure group most suitable for the calculation of the impact on human health. Human health risk quotients were calculated similar to those for the aquatic environment: as the ratio between the average daily exposure and API-specific HD_50_ values (*i*.*e*. the dose at which 50% of the individuals in 50% of mammalian species is being affected).

The environmental decision support system described by Oldenkamp *et al*. [17] compares two alternative pharmaceutical prescriptions on the basis of their Defined Daily Doses (DDDs). The study presented here, however, aims to estimate the environmental consequences of a shift in prescription behavior from prednisolone to berberine. However, DDDs have yet to be formulated for berberine. Therefore, the environmental impact of one DDD of prednisolone is compared with one DDD of berberine, assuming that their DDDs would be equal. This is done for each environmental grid cell throughout Europe. The ratio between the impacts of the two prescriptions then indicates how many times higher the actual DDD of berberine could be while still being an environmentally better alternative for prednisolone.

### Parameterization of the model

Substance-specific parameters were consistently parameterized according to a four-step preference approach:

1. Experimental or measurement data (green);
2. Extrapolation from related data (*e*.*g*. from degradation rates in other environmental media) (yellow);
3. Structure or property based predictions (*e*.*g*. the use of quantitative structure activity relationships (QSARs)) (orange);
4. Worst-case assumptions (red).

This enables an interpretation of the results through an analysis of the input data for prednisolone and berberine (Table 1). When important data gaps exist for prednisolone (*i*.*e*. worst-case assumptions are used), its environmental impact might be overestimated and the suitability of berberine as environmentally better alternative might then also be overestimated. Similarly, when important data gaps exist for berberine, the model might underestimate its suitability as environmentally better alternative for prednisolone.

**Table 1:** API-specific input parameters.

Parameter	Berberine			Prednisolone	
	Value [ref]	#		Value [ref]	#
*Physico-chemical characteristics*					
Vapor pressure (Pa)	1.4*10^−7 ^[36]	3		1.6*10^−12^ [37]	3
Water solubility (mg*L^-1^)	0.354 [38]	3		223 [39]	3
Octanol-water partitioning coefficient log_Kow_ (-)	2.1 [40]	3		1.62 [39]	1
*Human pharmacokinetic parameters*					
Excretion as parent compound (-)	0.00014 [41]	1		0.24 [22]	1
*Chemical fate during sewage treatment*					
k_bio,STP_ (h^-1^)	0	4		0.0070 [42]	1
N-removal efficiency (-)	0	4		0	4
P-removal efficiency (-)	0	4		0	4
UV-treatment removal (-)	0.32^a^ [43]	1		0	4
Ozonation removal (-)	0.50 [43]	1		0	4
Chlorination removal (-)	0	4		0	4
Sand filtration removal (-)	0	4		0	4
Microfiltration removal (-)	0	4		0	4
*Environmental fate*					
Biodegradation in water (s^-1^)	0	4		0	4
Photolysis in water (s^-1^)	0	4		4.15*10^−5^ [44]	1
Hydrolysis in water (s^-1^)	0 [45]	3		0	4
Biodegradation in soil (s^-1^)	0	4		0	4
Biodegradation in sediment (s^-1^)	0	4		0	4
*Human exposure–drinking water treatment*					
Conventional removal (-)	0.35 [46]	1^b^	4^b^	0	4
Advanced removal (-)	0.93 [46,47]	1^b^	4^b^	0	4
*Effect*					
Aquatic toxicity—HC_50_ (mg*L^-1^)	14.26 [48–54]	1		2.26 [44]	1
Mammalian toxicity—HD_50_ (mg*kgbw^-1^*d^-1^)	763.50 [55,56]	1		496.95 [57–59]	1

^a^ Calculated as the fraction removed during combined UV- and O_3_ treatment, minus the fraction removed after O_3_ alone

^b^ For the majority of drinking water treatments steps, worst-case no removal was assumed. For conventional treatment, these were the steps of coagulation, chlorination and sand filtration; for the advanced treatment, these were the steps of coagulation, chlorination, sand filtration, UV-treatment, ozonation, reverse osmosis and nanofiltration. Available information on removal during treatment with PAC (conventional and advanced treatment) and MBR (advanced treatment) were included in the calculations.

## Results

### Aquatic environment

**Fig 3:**
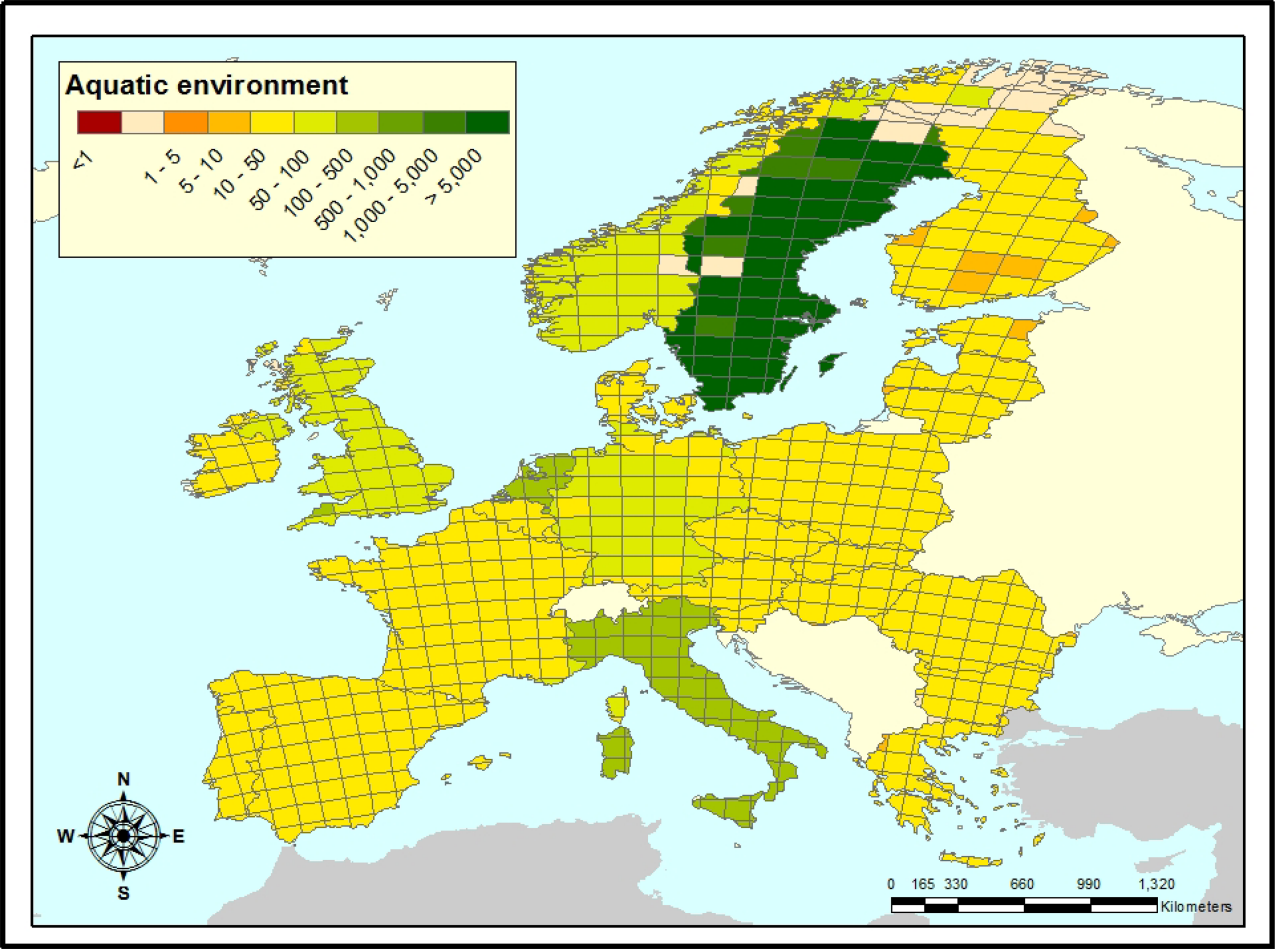
Ratios between the aquatic risk impacts of prednisolone and berberine in Europe. In white grid cells, the region is either sparsely populated without emission or are below 2000 p.e. (population equivalents) in size, which are not included in the model. In all grid cells prednisolone/berberine is larger than 1, which means that prescribing berberine is beneficial to prescribing prednisolone, even at a higher dosage. (Europe base map was generated using ArcMap [60].

The ratios between the aquatic impacts of prednisolone *vs*. berberine for Europe are given in Fig 3. A value of 1 indicates that, at equal dose, a prescription of berberine and a prescription of prednisolone have equal impacts on the aquatic environment. A value < 1 indicates that berberine might only be an environmentally beneficial alternative for prednisolone if its prescribed dose were lower. A value > 1 indicates that berberine could still be considered an environmentally beneficial alternative for prednisolone, even if it were prescribed at a higher dose.

In all grid cells throughout Europe, the ratio between aquatic impacts of prednisolone and berberine exceeds 1, with a median of 24.54 and 2.5^th^ percentile of 6.09 (Table 2). This means that, regardless of location, as long as the equivalent therapeutic dose of berberine is approximately 6 times that of prednisolone or less, this compound has less damaging impact on the aquatic environment and is the environmentally preferred choice for prescription. However, if the specific prescription location is known, the dosage of berberine might be up to three orders of magnitude higher than that of prednisolone, while still being beneficial.

**Table 2:** Ratios of impacts of prednisolone and berberine on the aquatic environment on infants’ health after consumption of water after conventional or advanced drinking water treatment (DWT). Sparsely populated grids without emissions are not taken into account in these calculations.

	Aquatic environment	Infants–conventional DWT	Infants–advanced DWT
Average	663.27	174.73	775.56
2.5 percentile	6.09	0.62	0.62
median	24.54	5.87	22.80

### Human health

**Fig 4:**
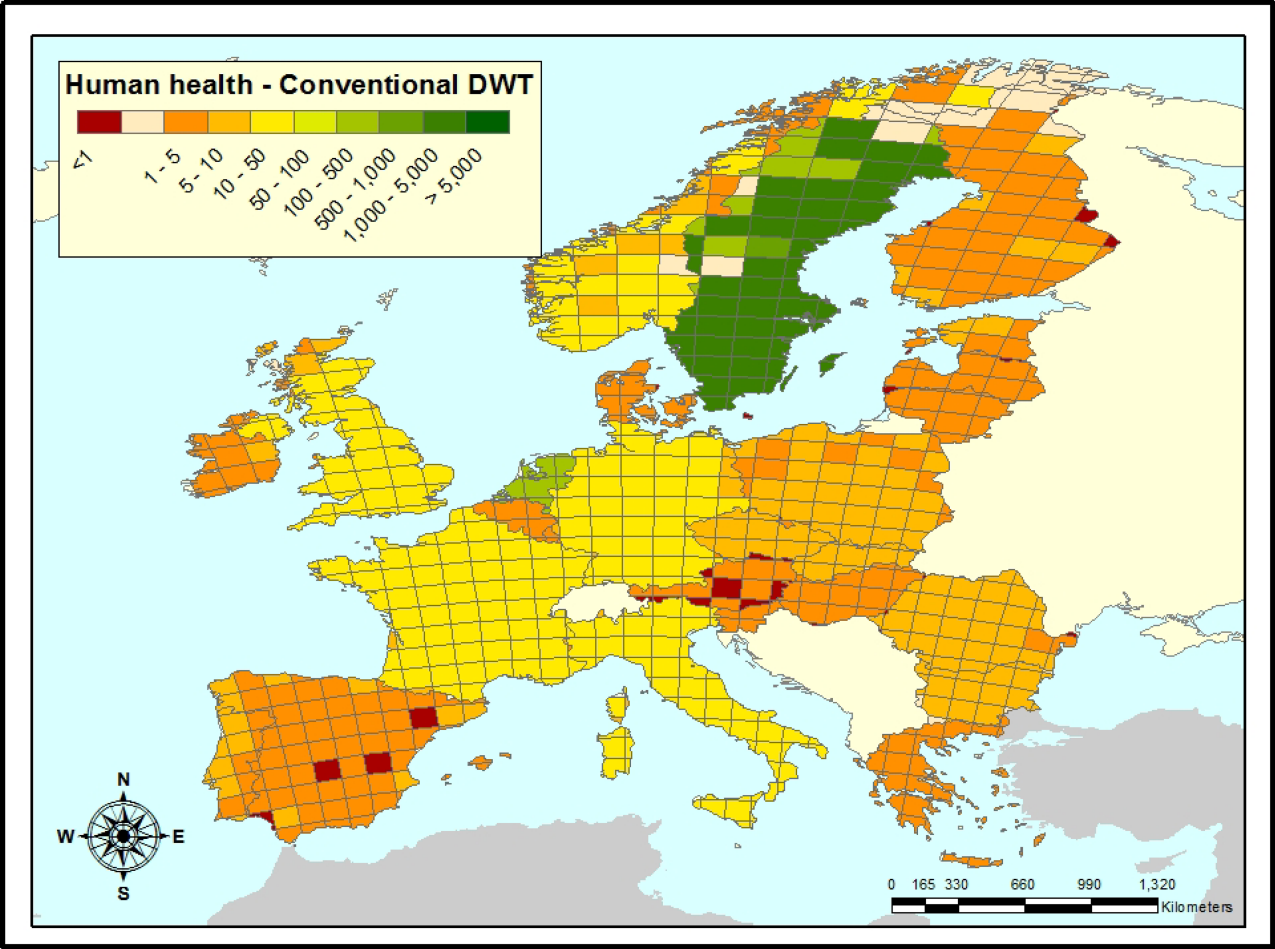
Ratios of human health impacts of prednisolone *vs*. berberine after conventional drinking water treatment. In white grid cells, the region is either sparsely populated without emission or are below 2000 p.e. (population equivalents) in size, which are not included in the model. Where prednisolone/berberine is smaller than 1 (red grid cells), berberine might only form an environmentally beneficial alternative for prednisolone, if its therapeutic dose would be lower. Where prednisolone/berberine is larger than 1, prescribing berberine is beneficial to prescribing prednisolone, even at higher dosage. (Europe base map was generated using ArcMap [60].

Similar to the aquatic environment, the health impact on infants after consumption of water after conventional drinking water treatment (DWT) is given as the ratio of impacts of prednisolone *vs*. berberine (Fig 4). In most EU member states, at equal dosage, berberine has a lower impact on infants' health than prednisolone with a median ratio of 5.87 (Table 2). This means that the therapeutic dose of berberine can be almost 6 times higher than prednisolone and still be beneficial. However, the 2.5^th^ percentile of the ratios throughout Europe is below 1 (0.61), meaning that for at least 1 in 40 grid cells, berberine would only be environmentally beneficial with a prescribed dose below that of prednisolone. This holds for some regions in Spain, Austria, Baltic States and Finland. Prescription of berberine over prednisolone is most beneficial in Sweden and The Netherlands.

**Fig 5:**
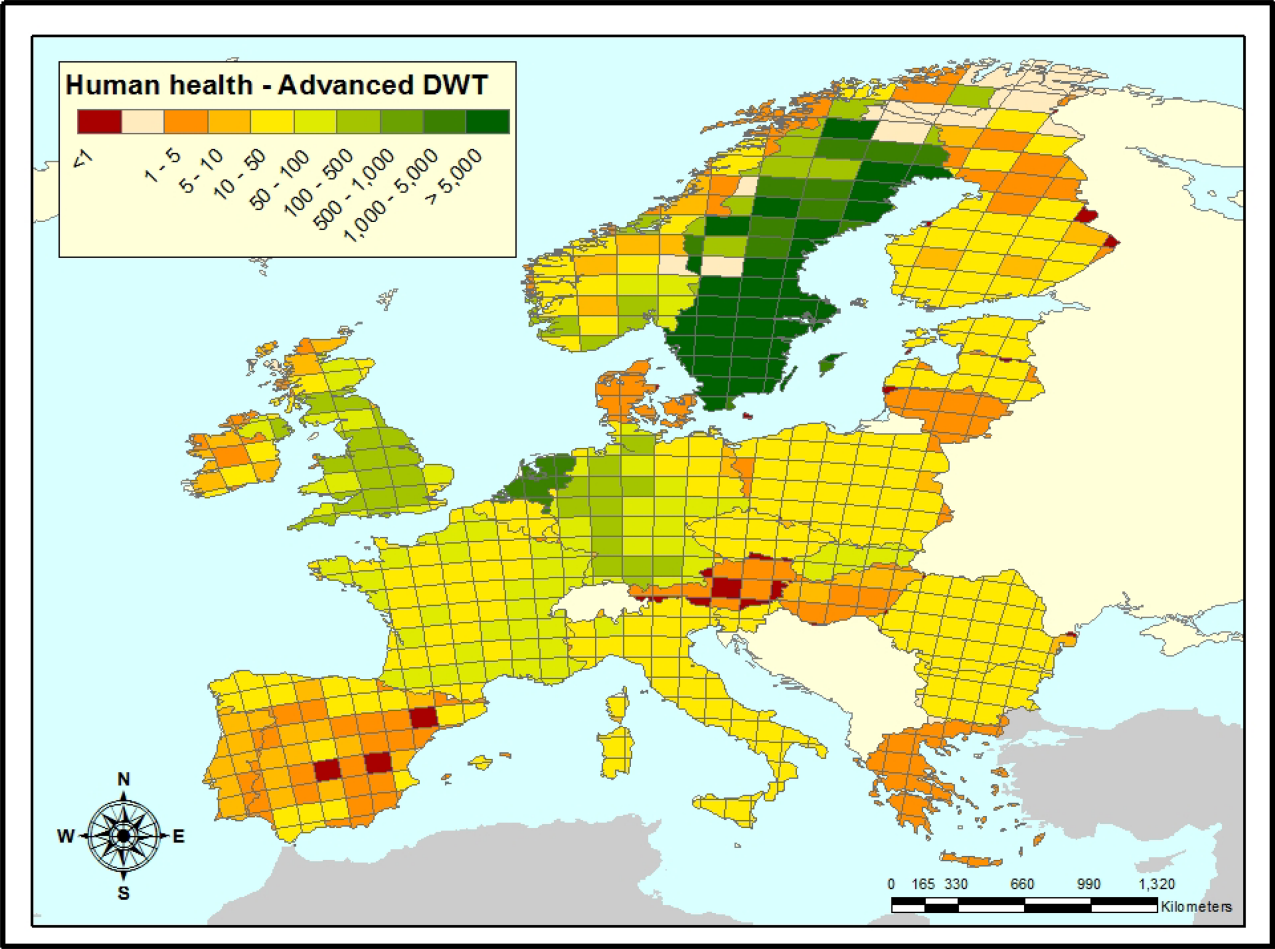
Ratios of human health impacts of prednisolone *vs*. berberine after advanced drinking water treatment. In white grid cells, the region is either sparsely populated without emission or are below 2000 p.e. (population equivalents) in size, which are not included in the model. Where prednisolone/berberine is smaller than 1 (red grid cells), berberine might only form an environmentally beneficial alternative for prednisolone, if its therapeutic dose would be lower. Where prednisolone/berberine is larger than 1, prescribing berberine is beneficial to prescribing prednisolone, even at higher dosage. (Europe base map was generated using ArcMap [60].

The impact on infants after consumption of water after advanced DWT is again lower for berberine than for prednisolone in most EU member states (Fig 5). The impact ratios even decreased throughout Europe compared with the conventional DWT scenario. Advanced water treatment enhanced the favoring position of berberine over prednisolone in all countries, although this is not deducible from Figs 4 and 5 for Austria. Again, Sweden and The Netherlands stand out. The therapeutic dose of berberine can now generally be 22.8 (median) times higher than prednisolone (Table 2).

## Discussion

Although several chemical substance regulation programs are in place (TSCA in the USA, REACH and ERA in Europe), these programs are incomplete when it comes to protection of the (aquatic) environment and human health due to exposure to this environment [12]. It is important to look beyond the impact of pharmaceuticals on individual patients and include the environmental impact as well, also because humans might be indirectly affected due to environmental exposure. Therefore, in this study, we modeled the environmental consequences of a shift in prescription behavior from the synthetic corticosteroid prednisolone to the natural product berberine using an environmental decision support system [17].

Since the actual therapeutic dose of berberine is yet unknown, it can in principle not be concluded that the environmental impact of a prescription of berberine is less severe than one of prednisolone, even at impact ratios > 1. However, as long as the ratio of the therapeutic doses of prednisolone/berberine does not exceed the ratios of their environmental impacts, berberine is indeed the environmentally friendly alternative. Similarly, berberine could still be the environmentally friendly alternative if the impact ratio < 1, provided that the therapeutic dose of berberine is then lower than that of prednisolone.

We showed that throughout Europe, the aquatic impact ratios of prednisolone/berberine > 1, with a median of 24.54 (Fig 3, Table 2). This means that as long as the therapeutic dose of berberine < 6 times the dose of prednisolone, it is indeed less damaging for the aquatic environment in 97.5% of the EU regions modeled.

With respect to its impact on infants’ health, berberine is an environmentally alternative for prednisolone up to a defined dose (respectively 5.5 and 22 times the dose of prednisolone, depending on the DWT level) except for some regions in Spain, Austria, The Baltic States, and Finland (Figs 4 and 5, Table 2).

There are two main explanations for the relatively low impact of berberine compared to prednisolone. The first is its very low excretion fraction. After consumption only 0.01% of berberine is excreted into the sewerage, which is approximately 2400 times lower than the 24% for prednisolone. Consequently, the normally negligible influence of non-compliance with treatment and subsequent direct disposal of leftover pharmaceuticals into the sewerage becomes large for berberine. This is reflected in the relatively high impact ratios for Sweden and the Netherlands compared with the other countries assessed (Figs 3–5). Because in these two countries leftover pharmaceuticals are disposed of relatively little *via* direct flushing through sink or toilet [61], berberine remains environmentally beneficial over prednisolone at higher doses.

The second main explanation for the relatively low impact of berberine lies in its estimated aquatic and human health effects which are approximately 6.3 and 1.5 times less toxic than prednisolone, respectively. It is important to note, however, that the HC_50_ and HD_50_ values used in the calculations are measures of acute toxicity. As such, they do not reflect effect concentrations relevant for the long-term chronic exposure we are interested in. They were thus applied under the assumption that the relative difference between berberine and prednisolone in terms of their acute effects is the same as the relative difference in terms of their chronic effects.

In order to predict the environmental impact of prednisolone and berberine, the model was fed with API-specific parameters (Table 1). To get to the most realistic results, experimental and calculated data should be used to parameterize the model. However, only few data were available for this and several worst-case assumptions had to be made to model the fate of both APIs during STP and DWT, and in the environment. Each of these worst-case assumptions might have caused an under- or overestimation of the actual impact ratio of prednisolone *vs*. berberine. Parameters for which prednisolone data were available, but for which worst-case assumptions had to be made for berberine, might have led to an underestimation of the actual impact ratios. For example, information on environmental photolysis of prednisolone was available, but for berberine a worst-case no degradation assumption had to be made. Since photodegradation is the most important environmental degradation pathway for various drugs [62], it is likely that in reality berberine is at least partly degraded under solar radiation. This thus has led to an overestimation of berberine concentrations and an underestimation of actual impact ratios. *Vice versa*, actual impact ratios might have been overestimated due to parameters for which berberine data were available but prednisolone data were not. The latter parameters are of specific interest in light of the validity of our model results. Indeed, filling knowledge gaps for berberine will even further increase impact ratios between the two APIs, while filling knowledge gaps for prednisolone might decrease them.

Based on the above, two modeling steps deserve attention. The first relates to the fate of prednisolone during tertiary (advanced) wastewater treatment. Berberine is removed to quite an extent during wastewater treatment with ozonation and UV radiation techniques (with removal efficiencies of 0.32 and 0.50, respectively; Table 1), while we assumed these techniques to have a worst-case zero removal efficiency for prednisolone. In reality, the efficiency of these techniques to remove prednisolone is potentially substantial, leading to an overestimation of the impact ratios for regions with STPs that apply these advanced STP techniques. However, UV-treatment and ozonation are only scarcely applied in European wastewater treatment (3% and 0.2% of all European STPs, respectively), limiting the overall influence of these worst-case assumptions. Moreover, the rate constant for biodegradation during (far more widely applied) secondary treatment was assumed zero for berberine, while this information was available for prednisolone (0.0070 h-1; Table 1). This further reduces the influence of these worst-case assumptions on the modeled impact ratios for prednisolone.

Removal during DWT is a second modeling step that deserves attention, because of the lack of information on the removal of prednisolone during DWT in comparison with berberine. Especially since human health impact ratios are <1 in some grid cells in Europe, the fate of prednisolone during DWT is a topic relevant for future research. It is, however, important to realize that drinking water is only one of multiple routes *via* which humans might be exposed to berberine and prednisolone. Consumption of food products such as vegetables, fish, meat and dairy products, form other potentially important routes of exposure. API residues adsorbed to sludge might become available for uptake in vegetables in countries where this sludge is (partly) applied to agricultural soils. Both the sorption to sewage sludge as well as transfer into vegetables, fish, and meat and dairy products increase with increasing K_OW_. Because berberine has a 1.3 times higher K_OW_ than prednisolone (Table 1), it also ends up more easily in aforementioned food products. This limits the relative importance of drinking water as exposure route, and consequently limits the influence of worst-case assumptions made for prednisolone during DWT.

Finally, it should be noted that environmental degradation processes were barely incorporated in the assessment, with the exception of the aforementioned photolysis of prednisolone, because data on these were almost entirely lacking for both prednisolone and berberine (Table 1). As a consequence, impact ratios hardly vary between grid-cells within one country and most spatial variability in impact ratios can be attributed to differences between countries, such as levels of non-compliance, leftover APIs disposal, and consumption behavior. Impact ratios for human health (Figs 4 and 5) show slightly more within-country variation than those for the aquatic environment (Fig 3). This relates to between-grid cell variation in emissions to agricultural soils and between-grid cell variation in environmental parameters influencing uptake into food products.

## Conclusion

This study was performed to raise awareness in the pharmaceutical community on the impact of pharmaceuticals on the (aquatic) environment and that changing from one drug to another could be beneficial for both aquatic as well as human health. As an example, the synthetic corticosteroid prednisolone was compared to the natural alkaloid berberine. We showed that in most regions in Europe, it is up until a certain dose of berberine, beneficial for both the aquatic environment as well as for human health to prescribe berberine over prednisolone. To strengthen the validity of these results, we strongly recommend further research into the fate of both APIs during wastewater and drinking water treatment. The filling of these knowledge gaps will decrease the extent of worst-case assumptions required in model parameterization, and as such will increase the confidence that can be attributed to the results. Additional to the attention for adverse effects in patients and socio-economic considerations, regulations on prescription of alternative drugs taking into account their environmental impact, should be encouraged.

## Abbreviations

API: active pharmaceutical ingredient
BCF: bioconcentration factor
BTF: biotransfer factor
DDD: defined daily dose
DWT: drinking water treatment
MBR: membrane bioreactor
PAC: powdered activated carbon
RCF: root concentration factor
STP: sewage treatment plant
QSAR: quantitative structure activity relationship.
